# Association between serum folate levels and migraine or severe headaches: A nationwide cross-sectional study

**DOI:** 10.1097/MD.0000000000040458

**Published:** 2024-11-08

**Authors:** Huang Luwen, Chen Ping, Ouyang Qing-Rong, Xu Lei, Li Linlin, Ming Yu

**Affiliations:** a Department of Neurology, Suining Central Hospital, Suining, Sichuan Province, China; b Department of Pharmacy, Suining Central Hospital, Suining, Sichuan Province, China.

**Keywords:** cross-section study, headache, migraine, NHANES, serum folate

## Abstract

Migraine and severe headaches are common neurological disorders with significant societal impact. Previous research indicates a potential link between serum folate levels and migraine occurrence, yet there is a lack of sufficient relevant studies and more are required. This study aimed to determine the association between a severe headache or migraine and serum folate levels in large populations. Using data from the National Health and Nutrition Examination Survey, we conducted a cross-sectional study. Using multivariable logistic regression models, we investigated the association between serum folate and severe headache or migraine. In a subsequent subgroup analysis, several confounding factors were also explored to investigate the association between migraine and serum folate. A total of 13,351 individuals participated in the study, with 2742 reporting severe headache or migraine in the previous 3 months. Serum folate was negatively associated with severe headache or migraine (odds ratio [OR] = 0.5, 95% confidence interval [CI] = 0.28–0.89, *P* = .018). The stratified analysis revealed this association persisted among female (OR = 0.38, 95% CI = 0.18–0.82, *P* < .001), individuals aged 20 to 50 years (OR = 0.53, 95% CI = 0.28–0.99, *P* < .001), and non-Hispanic White participants (OR = 0.38, 95% CI = 0.17–0.87, *P* < .001). We found that greater levels of serum folate were significantly related to a decreased likelihood of migraine onset, especially among women, young and middle-aged populations, and non-Hispanic White participants. Further research is required to validate and expand upon our results.

## 1. Introduction

Migraine, a common neurovascular disorder of unknown etiology, is considered a disabling neurological condition characterized by recurrent headaches, nausea, vomiting, sensitivity to light and sound, and phonophobia.^[1]^ In the United States, migraine had a prevalence of approximately 18.2% among females and 6.5% among males, with the highest occurrence observed in individuals aged 25 to 55.^[2]^ Migraine results in a significant decrease in the quality of life and incur heavy costs for sufferers.^[3]^

Currently, migraine is regarded as a complex neuroinflammatory disorder with predominant activation of the trigeminovascular system, yet its molecular mechanisms remain unclear.^[4,5]^ Migraine may be influenced by the following metabolic factors: behavioral, environmental, dietary, hormonal, and genetic.^[5]^ Hyperhomocysteinemia (HHcy) is a widely recognized risk factor associated with neurological disorders, cognitive decline, and cardiovascular disease.^[6]^ Smith AD and colleagues discovered a correlation between serum homocysteine (Hcy) level and the frequency and characteristics of headache attacks.^[7]^ HHcy may induce migraine as it enhances neuronal excitability, triggering the release of various headache mediators and inflammatory factors.^[8]^ Folic acid, a water-soluble vitamin, plays a significant role in Hcy metabolism by promoting its methylation reaction.^[9]^ Recently, it was reported that folic acid supplementation can alleviate migraine symptoms by affecting Hcy levels.^[10]^

Nevertheless, the association between serum folate and migraine is uncertain. This study is the first to demonstrate the relationship in a large population sample sourced from the National Health and Nutrition Examination Survey (NHANES). Furthermore, this study investigates the dose-effect correlation of serum folate in migraine.

## 2. Materials and methods

### 2.1. Study population

**Figure 1.**
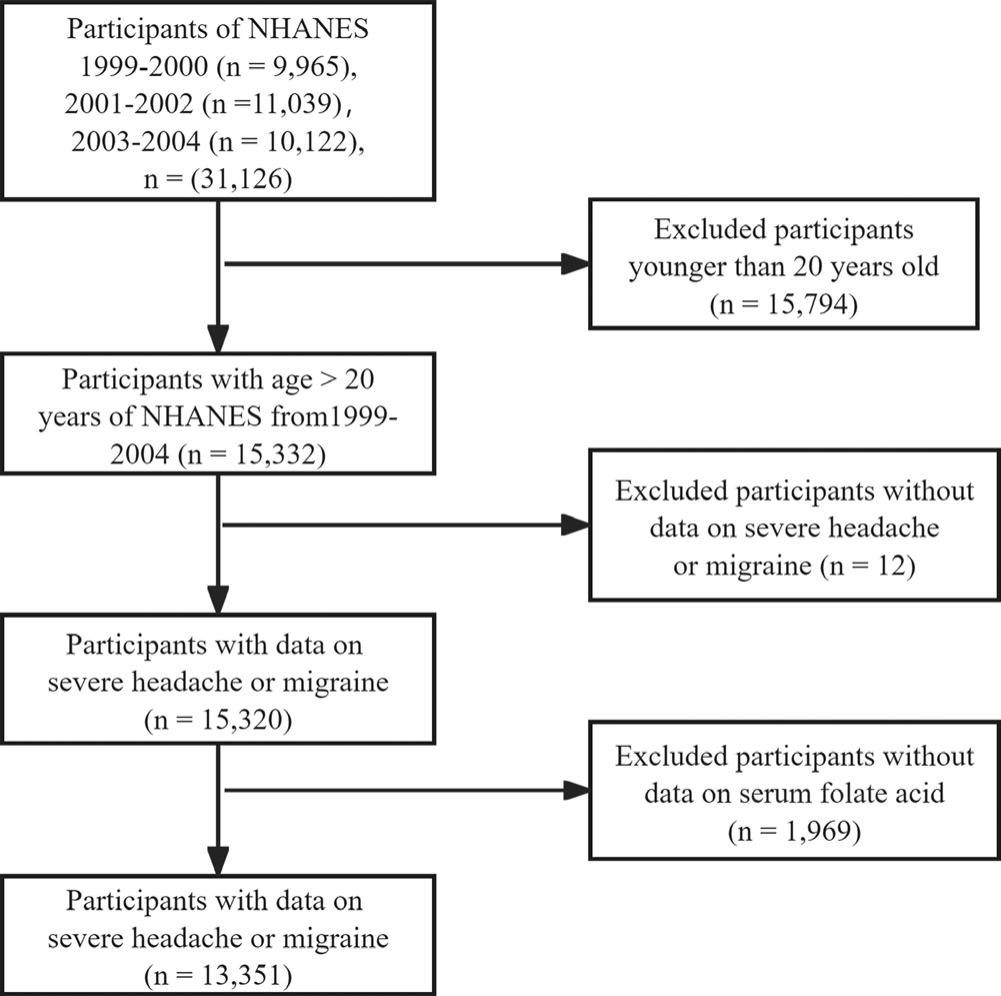
Detailed information about subject recruitment.

The data analyzed in this study were obtained from NHANES (1999–2004), comprising a total of 31,126 individuals. Participants were excluded based on the following criteria: (1) individuals under 20 years of age (n = 15,794); (2) those without data on severe headache or migraine (n = 12); and (3) those without serum folate measurements (n = 1969). Ultimately, 13,351 participants were included in the secondary analysis. Detailed flowchart about subject recruitment is illustrated in Figure 1. The NHANES Institutional Review Board approved the ethical conduct of NHANES 1999–2004 (Protocol#98-12), and all participants provided informed consent.

### 2.2. Headache assessment

Severe headache or migraine were evaluated via self-report in the miscellaneous pain section of the NHANES questionnaire. Participants who answered “yes” to the question regarding experiencing severe headache or migraine in the last 3 months were categorized as having such conditions. The results of the AMPP study support our hypothesis.^[11]^ According to the study, 17.4% of participants reported experiencing severe headache. Among them, 11.8% met the criteria for migraine as outlined in the International Headache Disorder Type II, 4.6% met the criteria for possible migraine, and only 1% fell into the category of other severe headache. It is reasonable to assume that the majority of participants reporting severe headache are experiencing migraine.

### 2.3. Exposure variable

Serum folate served as the exposure variable in this cross-sectional study. It was measured using the “Quanta phase II Folate” radio assay kit from Bio-Rad Laboratories, as detailed on the NHANES website. Serum folate was categorized into 3 groups: Q1 (deficiency, <3.1 ng/mL), Q2 (lower normal range, 3.1–17.5 ng/mL), and Q3 (higher normal range, >17.5 ng/mL).^[12]^

Covariates were selected based on biological factors and existing literature, while demographic information, including age, sex, height, weight, education level, race/ethnicity, smoking and drinking status, and family income-to-poverty ratio, was collected via a standardized questionnaire. The body mass index (BMI) was calculated based on self-reported weight and height. Education levels were classified into 3 groups: below high school, high school, and above high school. Participants were classified as smokers or nonsmokers based on their smoking status, and drinking status was defined as consuming at least 12 alcoholic drinks in the previous 12 months.^[13]^ A family income-to-poverty below 1 indicates poverty.

Furthermore, laboratory tests were performed to evaluate serum levels of total cholesterol, triglycerides, low-density lipoprotein cholesterol, high-density lipoprotein cholesterol, C-reactive protein (CRP), Hcy, and vitamin B12. The definition of hyperlipidemia met one of the following criteria: (1) triglycerides ≥ 150 mg/dL (1.7 mmol/L); (2) total cholesterol ≥ 200 mg/dL (5.18 mmol/L); (3) low-density lipoprotein cholesterol ≥ 130 mg/dL (3.37 mmol/L); (4) high-density lipoprotein cholesterol < 40 mg/dL (1.04 mmol/L) for males and < 50 mg/dL (1.3 mmol/L) for females; or (5) inclusion of participants using lipid-lowering medication.^[14]^ The serum Hcy levels were divided into 2 groups: normal (Hcy ≤ 15 µmol/L) and HHcy (Hcy > 15 µmol/L).^[15]^ Detailed descriptions of these covariates are available on the NHANES website.

### 2.4. Processing missing data

Simple replacement and dummy variables were used for categorical variables with missing data. For continuous variables with missing data, we used K-nearest neighbors interpolation.

### 2.5. Statistical analysis

Continuous data were summarized as mean ± standard deviation (mean ± SD) or mean ± interquartile range, and differences between the 2 groups were compared using the independent samples *t* test. Categorical data were presented as frequencies and percentages (n, %), and differences between groups were compared using the Pearson chi-square (χ^2^) test.

Logistic regression models were used to assess the independent correlation between severe headache or migraine and serum folate, both before and after adjustment for confounding factors. The results are shown with adjusted odds ratios (ORs) and 95% confidence intervals (CIs). Restricted cubic spline regression investigated the relationship between serum folate and severe headache or migraine. Following this, subgroup analyses were conducted stratified by sex, age, race/ethnicity, education, and poverty to examine the association between serum folate and severe headache or migraine in further detail.

Propensity score matching^[16]^ was used to mitigate the effects of confounding variables that could introduce outcome bias. The propensity score was calculated through a multivariate logistic regression model, using a participant’s serum folate. We used a 1:1 nearest neighbor matching algorithm with a caliper width of 0.2. An absolute SMD (standardized mean difference) <0.10 was considered indicative of a balanced PSM. The pairwise algorithmic and overlap weight models were used to generate a weighted cohort.^[17]^

We conducted all analyses using the statistical software packages R 3.3.2 (available at http://www.R-project.org, The R Foundation) and Free Statistics software, version 1.4. A 2-tailed test was employed, and statistical significance was defined as *P* < .05.

## 3. Results

### 3.1. Baseline characteristics of participants

Of the 2742 (20.54%) patients diagnosed with severe headache or migraine, the median age was 41.0 (30.0–54.0) years, with the control group having a median age of 51.0 (34.0–68.0) years. Meanwhile, the severe headache or migraine group consisted of 67.9% female and 48.4% male in the control group. There were statistically significant differences in the median age and gender between the 2 groups. Moreover, statistically significant differences were observed in either race distribution (*P* < .001) or education level (*P* < .001). Furthermore, the severe headache or migraine group showed lower serum folate levels (11.6 vs 12.7, *P* < .001) compared to the control group, while also demonstrating higher levels of CRP (0.3 vs 0.2, *P* < .001) and BMI (27.9 vs 27.3, *P* < .001). However, no significant differences were observed in serum vitamin B12 or hyperlipidemia. The data are shown in Table 1. Table S1, Supplemental Digital Content, http://links.lww.com/MD/N871 illustrates that female (OR = 2.26, 95% CI = 2.07–2.47, *P* < .001) and unmarried status (OR = 1.26, 95% CI = 1.13–1.41, *P* < .001) were positively associated with severe headache or migraine. Conversely, age 50 to 85 years (OR = 0.44, 95% CI = 0.41–0.49, *P* < .001), non-Hispanic White (OR = 0.79, 95% CI = 0.71–0.87, *P* < .001), above high school (OR = 0.78, 95% CI = 0.71–0.86, *P* < .001), absence of poverty (OR = 0.62, 95% CI = 0.56–0.69, *P* < .001), high serum folate levels (Q3 > 17.5ng/mL) (OR = 0.41, 95% CI = 0.24–0.72, *P* = .002), and high Hcy levels (OR = 0.93, 95% CI = 0.92–0.95, *P* < .001) were negatively associated with severe headache or migraine.

**Table 1:** Characteristics of participants with or without headache.

Characteristic	Total (n = 13,351)	Non-headache (n = 10609)	Severe headache or migraine (n = 2742)	*P*
Sex, n (%)				<.001
Male	6353 (47.6)	5474 (51.6)	879 (32.1)	
Female	6998 (52.4)	5135 (48.4)	1863 (67.9)	
Age, mean (SD), years	48.0 (33.0, 66.0)	51.0 (34.0, 68.0)	41.0 (30.0, 54.0)	<.001
Age, n (%)				
20–50, years	7167 (53.7)	5275 (49.7)	1892 (69.0)	
50–85, years	6184 (46.3)	5334 (50.3)	850 (31.0)	
Race/ethnicity, n (%)				<.001
Mexican American	3039 (22.8)	2363 (22.3)	676 (24.7)	
Other Hispanic	606 (4.5)	448 (4.2)	158 (5.8)	
Non-Hispanic White	6759 (50.6)	5517 (52.0)	1242 (45.3)	
Non-Hispanic Black	2474 (18.5)	1912 (18.0)	562 (20.5)	
Other race	473 (3.5)	369 (3.5)	104 (3.8)	
Marital status, n (%)				<.001
Married or living with a partner	10,861 (84.2)	8703 (84.9)	2158 (81.6)	
Unmarried	2035 (15.8)	1548 (15.1)	487 (18.4)	
Education, n (%)				<.001
Below high school	4310 (32.3)	3329 (31.4)	981 (35.8)	
High school	3161 (23.7)	2497 (23.6)	664 (24.3)	
Above high school	5853 (43.9)	4761 (45)	1092 (39.9)	
Poverty, n (%)				<.001
Yes	2250 (18.4)	1632 (16.8)	618 (24.6)	
No	9976 (81.6)	8085 (83.2)	1891 (75.4)	
Alcohol intake, n (%)				<.001
Yes	8463 (67.8)	6874 (69.2)	1589 (62.5)	
No	4011 (32.2)	3058 (30.8)	953 (37.5)	
Hyperlipidemia, n (%)				.192
Yes	8849 (89.4)	7060 (89.2)	1789 (90.2)	
No	1048 (10.6)	854 (10.8)	194 (9.8)	
Smoking status, n (%)				<.001
Yes	1903 (44.8)	1429 (42.2)	474 (54.9)	
No	2347 (55.2)	1958 (57.8)	389 (45.1)	
Serum folate, n (%)				<.001
Q1 (<3.1 ng/mL)	62 (0.5)	43 (0.4)	19 (0.7)	
Q2 (3.1–17.5 ng/mL)	9841 (73.7)	7652 (72.1)	2189 (79.8)	
Q3 (>17.5 ng/mL)	3448 (25.8)	2914 (27.5)	534 (19.5)	
Serum folate (ng/mL) [median (IQR)]	12.4 (8.8, 17.8)	12.7 (9.0, 18.2)	11.6 (8.3, 16.2)	<.001
Folate supplement (ug) [median (IQR)]	17.5 (3.0, 49.0)	18.0 (4.0, 49.4)	16.0 (2.0, 46.0)	.005
Serum vitamin B12 (pg/mL) [median (IQR)]	464.0 (351.0, 613.0)	466.0 (351.0, 615.0)	457.5 (351.0, 603.8)	.188
Hcy (µmol/L) [median (IQR)]	7.9 (6.3, 10.0)	8.1 (6.5, 10.2)	7.2 (5.8, 9.1)	<.001
CRP (mg/dL) [median (IQR)]	0.2 (0.1, 0.5)	0.2 (0.1, 0.5)	0.3 (0.1, 0.6)	<.001
BMI [median (IQR)]	27.4 (24.1, 31.4)	27.3 (24.1, 31.2)	27.9 (24.0, 32.5)	<.001

BMI = body mass index, CI = confidence interval, CRP = C-reactive protein, Hcy = homocysteine, IQR = interquartile range, OR = odds ratio, SD = standard deviation.

During data cleaning, missing values were identified as follows: 27/13,351 (0.2%) for education level, 27/13,351 (0.2%) for education, 375/13,351 (2.8%) for BMI, 877/13,351 (6.6%) for drinking, 1125/13,351 (8.4%) for poverty, 3454/13,351 (25.9%) for hyperlipidemia, and 4501/13,351 (33.7%) for Hcy.

### 3.2. Association of severe headache or migraine with serum folate

**Figure 2.**
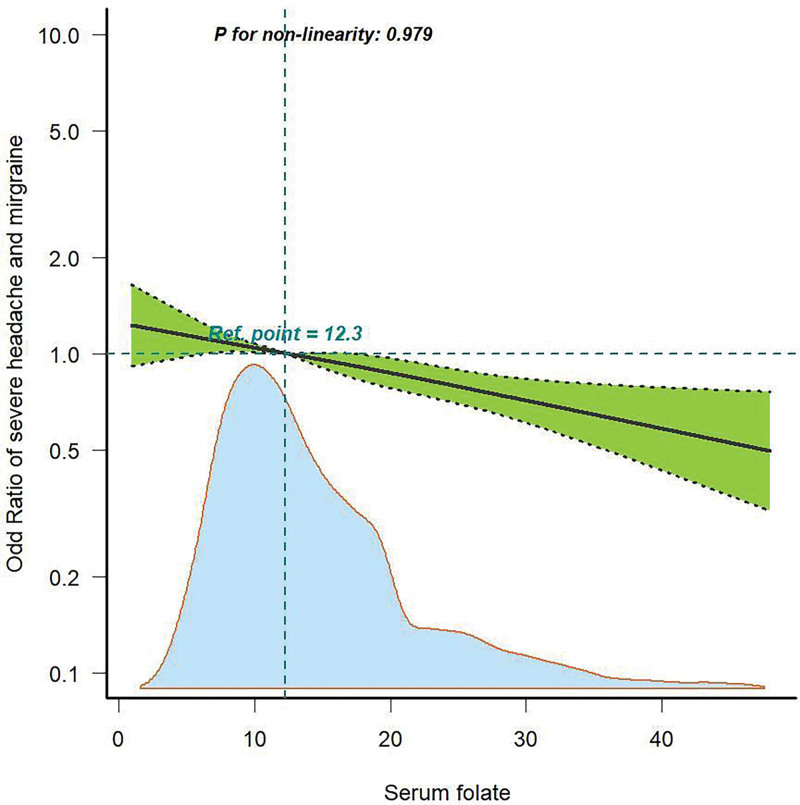
Association between serum folate level and severe headache or migraine in RCS. The model was adjusted for sex, age, race, marital status, poverty, education, hyperlipidemia, drinking, smoking, BMI, Hcy, CRP, serum folate acid supplement, and serum vitamin B12. Solid line, OR; shade, 95% CI. BMI = body mass index, CI = confidence interval, CRP = C-reactive protein, Hcy = homocysteine, OR = odds ratio, RCS = restricted cubic spline.

As displayed in Table 2, multivariable logistic regression analysis was conducted to investigate the association between severe headache or migraine and serum folate. In all 3 regression models (Models 1–3), a lower risk of severe headache or migraine was associated with high levels of serum folate (>17.5 ng/mL) (all *P* < .05). After fully adjusting for potential confounders, including sex, age, race, marital status, poverty, education level, hyperlipidemia, alcohol intake, smoking status, BMI, Hcy, CRP, folate acid supplementation, and serum vitamin B12 levels, the OR (95% CI) for the risk of severe headache or migraine in the Q3 group of serum folate was 0.5 (0.28–0.89) (*P* = .018). The results of Table 2 were consistent with those of Table S2, Supplemental Digital Content, http://links.lww.com/MD/N871 which employed a different missing data analysis approach. No evidence of a nonlinear relationship between serum folate level and severe headache or migraine was found (*P* = .797), suggesting that the risk of severe headache or migraine decreased with serum folate levels increasing (Fig. 2).

**Table 2:** Association between serum folate level and severe headache or migraine (dealing with missing values through dummy variables and KNN).

Characteristic	N	Model 1		Model 2		Model 3	
		OR (95%CI)	*P*	OR (95% CI)	*P*	OR (95% CI)	*P*
Serum folate acid	13,351	0.86 (0.81–0.91)	<.001	0.87 (0.82–0.92)	<.001	0.87 (0.82–0.92)	<.001
Serum folate acid							
Q1 (<3.1 ng/mL)	62	1 (Ref)		1 (Ref)		1 (Ref)	
Q2 (3.1–17.5 ng/mL)	9841	0.69 (0.39–1.2)	.191	0.70 (0.40–1.22)	.205	0.67 (0.38–1.19)	.173
Q3 (>17.5 ng/mL)	3448	0.5 (0.28–0.88)	.017	0.51 (0.29–0.91)	.022	0.5 (0.28–0.89)	.018

Model 1 was adjusted for sex, age, race, marital status, poverty, and education.

Model 2 was adjusted for Model 1 + hyperlipidemia, alcohol intake, smoking status, and BMI.

Model 3 was adjusted for Model 2 + Hcy, CRP, folate acid supplement, and serum vitamin B12.

BMI = body mass index, CI = confidence interval, KNN = K-nearest neighbors, OR = odds ratio, Ref = reference.

### 3.3. Subgroup analysis

**Figure 3.**
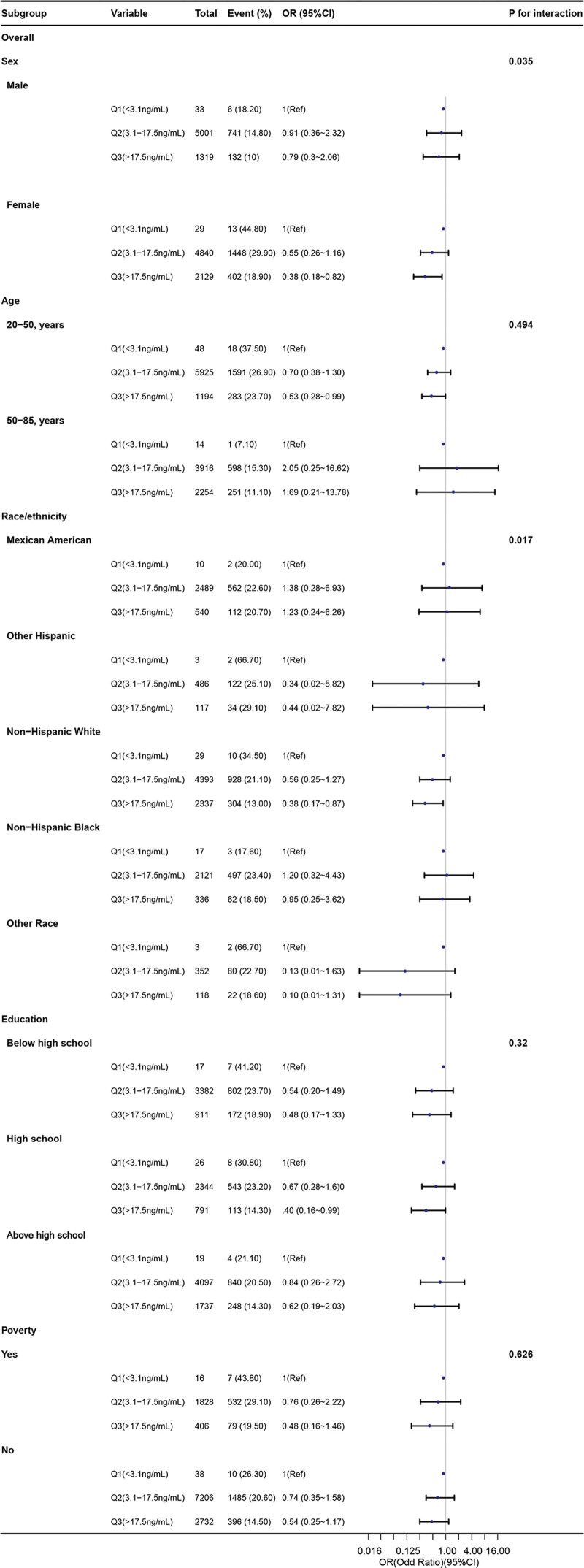
The forest plot shows ORs for subgroup analysis for the associations of serum folate and severe headache or migraine. The model was adjusted for sex, age, race, marital status, poverty, education, hyperlipidemia, drinking, smoking, BMI, Hcy, CRP, serum folate acid supplement, and serum vitamin B12. BMI = body mass index, CRP = C-reactive protein, Hcy = homocysteine, OR = odds ratio.

Subgroup analysis based on sex, age, race/ethnicity, education, and poverty was conducted, as illustrated in Figure 3. Subgroup analyses, stratified by sex (*P* for interaction = .035) and race/ethnicity (*P* for interaction = 0.017), showed a statistically significant association between severe headache or migraine and high levels of serum folate. Among participants in the Q3 group of serum folate, female (OR = 0.38, 95% CI = 0.18–0.82, *P* < .001), aged 20 to 50 years (OR = 0.53, 95% CI = 0.28–0.99, *P* < .001), or non-Hispanic White individuals (OR = 0.38, 95% CI = 0.17–0.87, *P* < .001) were more likely to have a lower risk of migraine or severe headache compared to those in the Q1 group.

### 3.4. Sensitivity analysis

**Figure 4.**
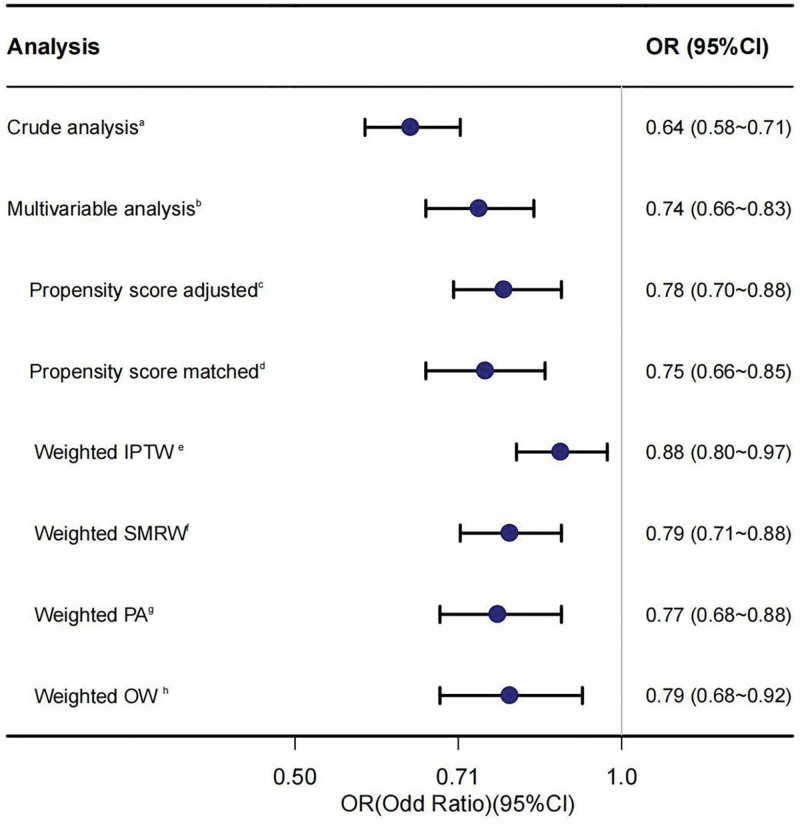
Forest plot shows ORs of serum folate level in severe headache or migraine using propensity score analysis. The model was adjusted for sex, age, race, marital status, poverty, education, hyperlipidemia, drinking, smoking, BMI, Hcy, CRP, serum folate acid supplement, and serum vitamin B12. BMI = body mass index, CRP = C-reactive protein, Hcy = homocysteine, OR = odds ratio.

To better analyze the association between high serum folate levels and severe headache or migraine, we merged the Q1 and Q2 groups of serum folate for sensitivity analysis. In the full cohort, even after adjusting for all significant covariates, propensity score analysis consistently indicated that high serum folate level were associated with a reduced risk of severe headache or migraine (OR = 0.78; 95% CI = 0.70–0.88, *P* < .001) (Fig. 4). Furthermore, when we applied inverse probability of treatment weighting (IPTW) in univariable modified logistic regression analysis, the OR remained consistent (OR = 0.88; 95% CI = 0.8–0.97, *P* < .001). Similarly, the ORs obtained from the weighted standardized mortality ratio weighting model, weighted pairwise algorithmic model, and weighted overlap weight model remained stable.

## 4. Discussion

Our cross-sectional study analyzed data from 13,351 participants in NHANES 1999 to 2004. A positive association was found between severe headache or migraine and high serum folate levels in a nationally representative US population, after adjusting for various confounding factors including sex, age, race, marital status, socioeconomic status, education level, hyperlipidemia, alcohol consumption, smoking status, BMI, Hcy, CRP, folate acid supplementation, and serum vitamin B12 levels. Subsequent subgroup analysis reveals that ORs for the risk of severe headache or migraine were lower among females, aged 20 to 50 years, and non-Hispanic White individuals in the high serum folate group.

There is considerable interest in interventions with serum folate to prevent severe headaches or migraines. Ferraris et al and a recent meta-analysis investigated the link between serum folate levels and migraine, discovering lower levels in migraine patients than in controls, which was in line with the findings in our study.^[18,19]^ Folic acid supplementation can reduce the frequency and severity of migraine. Additionally, high serum folate levels may prevent migraine.^[6,10]^ Previous studies have indicated that DNA methylation may be a potential factor contributing to the increased risk of migraines, facilitating the transition of episodic headaches to chronic headaches in migraine patients.^[20–23]^ Folate plays a crucial role in the synthesis of S-adenosylmethionine, which serves as a key methyl donor for DNA methylation.^[24–26]^ In addition, Inhibition of methionine production by low serum folate can result in the accumulation of Hcy in the body, causing HHcy. High levels of Hcy contribute to the risk of migraine through various mechanisms.^[19]^ Elevated levels of Hcy may injury to endothelial cells, reduced flexibility of the vessels, inhibiting cerebral cortex activity, and reducing local blood flow, which can lead to migraine.^[27]^ Furthermore, phosphorus nuclear magnetic resonance spectroscopy provides insights into alterations in brain energy metabolism among migraine patients, emphasizing the significance of an imbalance between brain energy demand and adenosine triphosphate production in migraine.^[28]^ This relationship underscores the role of mitochondrial function in the etiology of migraine.^[29]^ A study on familial hemiplegic migraine revealed that migraine individuals showed impaired energy metabolism in both the brain and muscles.^[30]^ Evidence has shown that mitochondrial dysfunction and impaired energy production in the central nervous system are attributed to high levels of Hcy.^[31]^ Currently, many studies have observed that high serum folate levels can reduce Hcy levels.^[32–34]^ Therefore, these findings suggest that reducing serum Hcy levels through high serum folate levels maintains normal mitochondrial function, potentially reducing migraine risk.

However, some reports indicated that no significant difference in serum folate levels has been observed between migraine and healthy populations.^[18,35,36]^ The small sample sizes or case series in existing research may affect the results. No specific studies have investigated the association between serum folate and migraine in large populations. Therefore, the data from NHANES offers an opportunity to better investigate the potential relationship between serum folate and severe headache or migraine in a substantial population, which makes the results more reliable.

Most study demonstrated that women were more likely to experience migraines than men,^[37]^ which is consistent with our findings. According to 1 prevalent theory, fluctuations in female sex hormones play a pivotal role.^[37]^ Serotonin, recognized for its crucial role in migraine pathogenesis, can be boosted by estrogen. Estrogen increases tryptophan hydroxylase expression and reduces serotonin reuptake transporter expression.^[38]^ Estrogen can also activate the endogenous opioid system, leading to an analgesic effect.^[39,40]^ Interestingly, it was discovered that the regional distribution of 5-methyltetrahydrofolate in the brain resembles that of serotonin.^[41]^ Folate deficiency was associated with reduced serotonin activity, while supplementation with folic acid increased brain 5-hydroxytryptamine concentration.^[42–44]^ Although men produce small amounts of estrogen through the aromatization of androstenedione and testosterone, these levels are much lower than those in young women and remain relatively stable.^[45]^ After menopause, women’s estrogen levels stabilize, leading to fewer migraine compared to premenopausal women. Nonetheless, they still have a higher migraine risk than men, possibly due to increased susceptibility to anxiety and depression.^[46–48]^ A study indicates that folate can improve depressive and anxiety-like behaviors in adult rats.^[49]^ Therefore, subgroup analysis revealed that high serum folate levels were associated with a reduced risk of severe headache or migraine in women but not in men. Further research is needed to explore the underlying reasons for this finding.

Non-Hispanic whites with high serum folate levels have a lower risk of severe headaches or migraines, but the cause is not yet fully understood. One possible explanation is that the prevalence of the methylenetetrahydrofolate reductase (MTHFR) TT gene variant is higher among non-Hispanic whites compared to various ethnic groups in the United States.^[50,51]^ MTHFR is a crucial enzyme in the body’s Hcy metabolism. The mutation of MTHFR C677T, occurring in approximately 12% of non-Hispanic whites, has been shown to increase plasma Hcy concentration and decrease serum folate levels.^[52,53]^ The CT and TT genotypes exhibit approximately 65% and 30% of MTHFR enzyme activity, respectively, compared to the CC genotype.^[54]^ Individuals carrying the C allele of the MTHFR C677T gene tended to have lower Hcy levels and a reduced risk of migraine compared to those with the TT genotype.^[27]^ Hence, high serum folate levels which reduce Hcy levels, may be associated with a lower risk of severe headaches or migraines in non-Hispanic whites.

Sun Yiyan et al reported that young and middle-aged populations tended to have higher levels of the Dietary Inflammatory Index.^[55]^ Higher levels of Dietary Inflammatory Index are associated with elevated levels of pro-inflammatory factors, including IL-1β, IL-1, 2, 4, 6, 10, tumor necrosis factor-α, and CRP, which significantly increase the likelihood of migraine onset.^[55–57]^ Recent studies have reported that folic acid shows remarkable anti-inflammatory effects and reduces levels of interleukins.^[58]^ Additionally, reports indicated that folic acid exhibits an antioxidant effect on oxidative stress, helping to alleviate cell damage.^[59]^ Consequently, it is plausible that individuals aged 20 to 50 may have a lower risk of severe headache or migraine with high serum folate levels.

Our study revealed a linear relationship where the risk of severe headache or migraine decreased with increasing serum folate levels. However, it does not imply that individuals can take high doses of folic acid to prevent migraine. Higher intake of folic acid might promote the growth of existing tumors, such as colon cancer, and induce genomic instability in peripheral lymphocytes.^[60–62]^. Nonetheless, some reports suggested that even at dosages of 15,000–100,000μg of folic acid daily, there was limited evidence of direct toxicity.^[63,64]^ Given the varying results from studies, extensive research is warranted to fully understand the appropriate dosage of folic acid to reduce the risk of migraine. According to previous reports, we currently recommend a folic acid dosage ranging from 0.5 to 2 mg.^[60–62]^

The limitations of this study were as follows: Establishing a causal relationship between migraine and serum folate is challenging due to the cross-sectional design. Therefore, future longitudinal investigations are necessary to draw causal inferences from current findings. Secondly, due to missing data in NHANES, it was not possible to distinguish between acute migraine attacks, the frequency and intensity of these attacks, and migraine with or without aura. Thirdly, despite our efforts to search the literature and control for potential confounders, it is important to recognize that migraine is a complex condition with various factors. Thus, additional confounders might not be accounted for in our study that could impact the risk of migraine.

## 5. Conclusion

Our study was the first to investigate the association between serum folate and severe headache or migraine in a large population sample. The results suggest that maintaining a high serum folate level may be crucial in preventing severe headache or migraine. As a recommendation, female, individuals aged 20 to 50 years, and non-Hispanic whites should pay more attention to their serum folate levels and consider folate intake if necessary.

## Author contributions

**Conceptualization:** Huang luwen, Chen Ping, Li Linlin, Ming Yu.

**Data curation:** Huang luwen, Ouyang Qing-rong, Ming Yu.

**Formal analysis:** Ouyang Qing-rong, Xu Lei, Li Linlin.

**Methodology:** Huang luwen, Chen Ping, Ouyang Qing-rong, Xu Lei, Ming Yu.

**Writing – original draft:** Huang luwen, Chen Ping.

**Writing – review & editing:** Li Linlin, Ming Yu.

## Supplementary Material


