# Supplementary material for: Association between serum folate levels and migraine or severe headaches: A nationwide cross-sectional study
Source: Medicine (Baltimore). 2024 Nov 8;103(45):e40458. doi: 10.1097/MD.0000000000040458 (PMC11557034; doi:10.1097/MD.0000000000040458)
Supplement: Supplementary file 1 [file medi-103-e40458-s001.pdf]

Supplementary Table 1

Relationship of variables and severe headache or migraine risk

Variable	OR (95% CI)	*p*-value
**Sex**		
Male	1(Ref)	
Female	2.26 (2.07~2.47)	<0.001
**Age**	0.98 (0.98~0.98)	<0.001
**Age, years**		
20-50	1(Ref)	
50-85	0.44 (0.41~0.49)	<0.001
**Race/ethnicity**		
Mexican American	1(Ref)	
Other Hispanic	1.23 (1.01~1.51)	0.041
Non-Hispanic White	0.79 (0.71~0.87)	<0.001
Non-Hispanic Black	1.03 (0.9~1.17)	0.676
Other Race	0.99 (0.78~1.24)	0.901
**Marital status**		
Married or living with partner	1(Ref)	
Unmarried	1.26 (1.13~1.41)	<0.001
**Education**		

Below high school	1(Ref)	
High school	0.9 (0.81~1.01)	0.071
Above high school	0.78 (0.71~0.86)	<0.001
**Poverty**		
Yes	1(Ref)	
No	0.62 (0.56~0.69)	<0.001
**Alcohol intake**		
Yes	1(Ref)	
No	1.32 (1.2~1.44)	<0.001
**Hyperlipidemia**		
Yes	1(Ref)	
No	0.87 (0.74~1.02)	0.091
**Smoking status**		
Yes	1(Ref)	
No	0.74 (0.66~0.83)	<0.001
**Serum folate**		
Q1 (＜3.1ng/mL)	1(Ref)	
Q2 (3.1-17.5ng/mL)	0.65 (0.38~1.11)	0.116
Q3 (＞17.5ng/mL)	0.41 (0.24~0.72)	0.002

**Folate acid supplement (ug)**	1 (0.999~1)	0.108
**Serum vitamin B12 (pg/mL)**	1 (1~1)	0.057
**Hcy (umol/L)**	0.93 (0.92~0.95)	<0.001
**CRP (mg/dL)**	1.06 (1.01~1.11)	0.011
**BMI**	1.02 (1.01~1.03)	<0.001

Abbreviations: BMI, body mass index; Hcy, Homocysteine; SD, standard deviation; IQR, interquartile range; CRP, C reactive protein.

Supplementary Table 2

Association between serum folate level and severe headache or migraine (Dealing with missing values through simple substitution and KNN).

Characteristic	N	Model 1		Model 2		Model 3	
		**OR (95%CI)**	**p**	**OR (95%CI)**	**p**	**OR (95%CI)**	**p**
**Serum folate acid**	13351	0.87 (0.82~0.92)	<0.001	0.87 (0.82~0.92)	<0.001	0.86 (0.81~0.91)	<0.001
**Serum folate acid**							
Q1 (< 3.1ng/mL)	62	1(Ref)		1(Ref)		1(Ref)	
Q2 (3.1-17.5ng/mL)	9841	0.69 (0.39~1.2)	0.187	0.69 (0.4~1.21)	0.2	0.67 (0.38~1.18)	0.169
Q3 (> 17.5ng/mL)	3448	0.5 (0.28~0.88)	0.016	0.51 (0.29~0.91)	0.022	0.50(0.28~ 0.89)	0.018

Model 1 was adjustd for sex,age, race, marital status, poverty and education;

Model 2 was adjusted for Model 1 + hyperlipidemia,alcohol intake,smoking status and BMI;

Model 3 was adjusted for Model 2 + Hcy,CRP, folate acid supplement and serum vitamin B12;

Abbreviations: OR, odds ratio ; CI, confidence interval.; Ref, reference.
